# Usefulness of Wide-Awake Local Anesthesia No Tourniquet Surgery to Decide on Tendon Transfer Versus Grafting in Chronic Flexor Tendon Rupture

**DOI:** 10.1016/j.jhsg.2022.04.001

**Published:** 2022-05-11

**Authors:** Mineyuki Zukawa, Ryusuke Osada, Tatsurou Hirokawa, Kayo Suzuki, Hiroto Makino, Yoshiharu Kawaguchi

**Affiliations:** ∗ Department of Orthopaedic Surgery, Faculty of Medicine, University of Toyama, Toyama, Japan

**Keywords:** Flexor digitorum profundus, Flexor pollicis longus, Tendon rupture, Wide-awake surgery

## Abstract

**Purpose:**

We investigated the clinical outcomes of flexor tendon reconstruction for chronic rupture of the flexor tendon based on an evaluation of the voluntary active contraction distance (ACD) of the ruptured musculotendinous unit and changes in intraoperative total active motion (TAM) that could only be observed during wide-awake local anesthesia no tourniquet (WALANT) surgery.

**Methods:**

Reconstructions of 19 tendons of the flexor pollicis longus (FPL) and 18 tendons of the flexor digitorum profundus (FDP) were performed during WALANT surgery to evaluate the ACD of the ruptured musculotendinous unit and TAM observed during the surgery. Tendon grafting or tendon transfer was selected during the surgery based on ACD. TAM, pinch strength, and grip power were evaluated before the surgery, during the surgery, and at final follow-up, and they were surveyed based on Quick Disabilities of the Arm, Shoulder, and Hand (q-DASH) scores. The final outcomes of tendon grafting and tendon transfer were compared.

**Results:**

In FPL tendon reconstruction, tendon grafting was performed in 10 patients with a total PDD and ACD value greater than 30 mm, and tendon transfer was performed in 9 patients with the value less than 30 mm. In FDP tendon reconstruction, tendon grafting was performed in 8 patients and tendon repair in 2 patients with a total PDD and ACD value greater than 40 mm, and tendon transfer was performed in 8 patients with the value less than 40 mm. The TAM value, q-DASH score, pinch power, and grip strength were improved in all patients. In both the tendon reconstructions, intraoperative TAM was significantly increased compared with preoperative TAM but significantly decreased at final follow-up. No significant differences were identified in final follow-up TAM and the q-DASH scores between tendon transfer and tendon grafting.

**Conclusions:**

The great advantage of WALANT surgery is that surgeons can evaluate the ruptured musculotendinous unit and measure TAM during the surgery.

**Type of study/level of evidence:**

Therapeutic I.

Chronic, closed, and spontaneous ruptures of the flexor pollicis longus (FPL) and flexor digitorum profundus (FDP) tendons differ from acute rupture and frequently result from blunt trauma, neglected injury, synovitis, and repetitive mechanical stress. The reconstruction of chronic rupture of the FPL tendon was previously usually performed using a bridging tendon graft or tendon transfer of the third or fourth flexor digitorum superficialis (FDS).^1^, ^2^, ^3^, ^4^, ^5^, ^6^, ^7^, ^8^, ^9^, ^10^ The reconstruction of chronic rupture of the FDP tendon is usually performed using a bridging tendon graft or tendon transfer of another FDP (side-to-side suture).^11^, ^12^, ^13^, ^14^, ^15^ The choice between tendon transfer and tendon grafting remains controversial and depends on the type of injury, time from rupture, and presence of a myostatic contracture. Traditionally, passive distraction distance (PDD) is a major determining factor.^16^^,^^17^ However, we consider active contraction distance (ACD) as the most important factor for determining function after tendon grafting because it directly contributes to the power source that moves the fingers.

The wide-awake local anesthesia no tourniquet (WALANT) technique has already gained widespread recognition and has been adopted as a useful technique for tendon surgery.^18^^,^^19^ This technique allows the surgeon to observe tendon excursion, adjust tension, and provide education to the patient. Furthermore, we have previously reported how to evaluate ruptured musculotendinous units during WALANT surgery.^20^^,^^21^ This new technique is useful for evaluating the musculotendinous unit for tendon reconstruction and allows the selection of adequate muscle, potentially contributing to better clinical outcomes. Thus, it may serve as an index for choosing between tendon grafting and tendon transfer.

Some reports have described better outcomes of the WALANT approach and overtensioning in tendon transfer from the extensor indicis proprius to the extensor pollicis longus, but no reports appear to have investigated flexor tendons.^22^, ^23^, ^24^ Furthermore, WALANT surgery reveals intraoperative total active motion (TAM), but descriptions of intraoperative TAM measurement and its trends remain lacking. The purpose of this study was to investigate the clinical outcomes of FPL and FDP tendon reconstruction during WALANT surgery by measuring ACD and intraoperative TAM that could only be observed during WALANT surgery. We also investigated factors affecting the results by investigating intraoperative TAM and its trends. We hypothesized that the measurement of intraoperative TAM would reveal the appropriate tension of suturing to determine whether standard tensioning with full range of intraoperative TAM or overtensioning, to account for loosening and adhesions, at the suture site is better.

## Materials and Methods

This study was approved by the ethics committee at the University of Toyama (Toyama, Japan) (clinical research number 21-22). Informed consent was obtained from each patient.

### FPL tendon reconstruction using the WALANT approach

Nineteen consecutive patients with chronic tendon rupture of FPL underwent tendon reconstruction during WALANT surgery at our department from April 2011 to June 2021. The study group comprised 9 men and 10 women, with a mean age of 66 ± 12 years (range, 32–77 years). The causes of tendon rupture were volar plate fixation due to a distal radius facture in 9 cases, rheumatoid arthritis in 5 cases, old rupture due to trauma in 3 cases, and trapeziometacarpal joint arthrodesis in 2 cases. The time from rupture to reconstruction ranged from 1 to 48 months (median, 3 months). The proximal stump of the ruptured FPL tendon was mobilized, and both PDD and ACD were measured (Video 1). If the total of PDD and ACD exceeded 30 mm, the ruptured musculotendinous unit was considered available for motor function, and tendon grafting was performed. In contrast, if the total of PDD and ACD was less than 30 mm, the ruptured musculotendinous unit was considered unavailable for motor function, and tendon transfer using the fourth FDS was performed. The PDD and ACD of the FDS tendon harvested as a donor tissue were measured in the same way as those of the FPL tendon. In both the procedures, 2 hand surgeons (M.Z. and R.O.) measured each distance. We investigated whether a correlation existed between PDD and ACD. In addition, we examined whether these distances were affected by the time elapsed prior to reconstruction. Furthermore, the suture tension was adjusted as standard tensioning to allow full range of intraoperative TAM for the early period in 9 patients and as overtensioning with full range of intraoperative flexion and limited extension for the later period in 4 patients.

The intraoperative active range of motion of the interphalangeal (IP) joint and the TAM of the thumb could be measured during WALANT surgery. Comparisons were made among the measurements made before the surgery (Video 2), during the surgery (Video 3), and at final follow-up (Video 4) to evaluate the outcomes. The follow-up duration ranged from 5 to 36 months (median, 12 months). The pinch strength was measured, and the patients were surveyed to determine the Quick Disabilities of the Arm, Shoulder, and Hand (*Quick*DASH) scores before the surgery and at final follow-up. The final outcomes of tendon grafting and tendon transfer were compared. In addition, the standard-tensioning and overtensioning groups were compared. Two cases were excluded from the comparisons of outcomes because of a joint contracture.

### FDP tendon reconstruction using the WALANT approach

A total of 18 consecutive fingers with chronic tendon rupture of FDP underwent tendon reconstruction during WALANT surgery at our department from April 2011 to June 2021. The study group comprised 6 men and 9 women, with a mean age of 73 ± 9 years (range, 49–89 years). Ten tendon reconstructions were performed in the index finger, 1 in the middle finger, 1 in the ring finger, and 6 in the little finger. The causes of tendon rupture were volar plate fixation of a distal radius facture in 4 cases, pyogenic tenosynovitis in 4 cases, rheumatoid arthritis in 3 cases, osteoarthritis in 3 cases, and old rupture due to trauma in 2 cases; the causes were unknown in 2 cases. The time from rupture to reconstruction ranged from 1 to 13 months (median, 2 months). Furthermore, the PDD and ACD of FDP were measured in the same way as those of FPL, as described above. If the total of PDD and ACD exceeded 40 mm, the ruptured musculotendinous unit was considered available for motor function, and tendon grafting or tendon repair was performed. In contrast, if the total of PDD and ACD was less than 40 mm, the ruptured musculotendinous unit was not considered to be available for motor function, and tendon transfer to another FDP (side-to-side suture) was performed. The suture tension was adjusted as standard tensioning with full range of intraoperative TAM for the early period in 8 fingers and as overtensioning with full range of intraoperative flexion and limited extension for the later period in 10 fingers. Because flexion lag often remained in the standard-tensioning group for the early period of the study, the overtensioning group for the later period was considered to have overtensioned suturing. The intraoperative TAM of the affected finger could be measured during WALANT surgery. Comparisons were made among the values measured before the surgery, during the surgery, and at final follow-up. The follow-up duration ranged from 3 to 36 months (median, 12 months). The grip power was measured, and the patients were subjectively surveyed to determine the *Quick*DASH scores before the surgery and at final follow-up. The final outcomes of tendon grafting and tendon transfer were compared.

### Statistical analysis

All data are shown as mean and SD. Correlation coefficients among PDD, ACD, and the time elapsed prior to reconstruction were calculated. Correlation analyses were performed using the Pearson correlation test. The Student *t* test was used to identify significant differences between the 2 groups. *P* values <.05 were considered statistically significant, and 95% confidence intervals were calculated for all analyses.

## Results

### FPL tendon reconstruction using the WALANT approach

**Figure 1:**
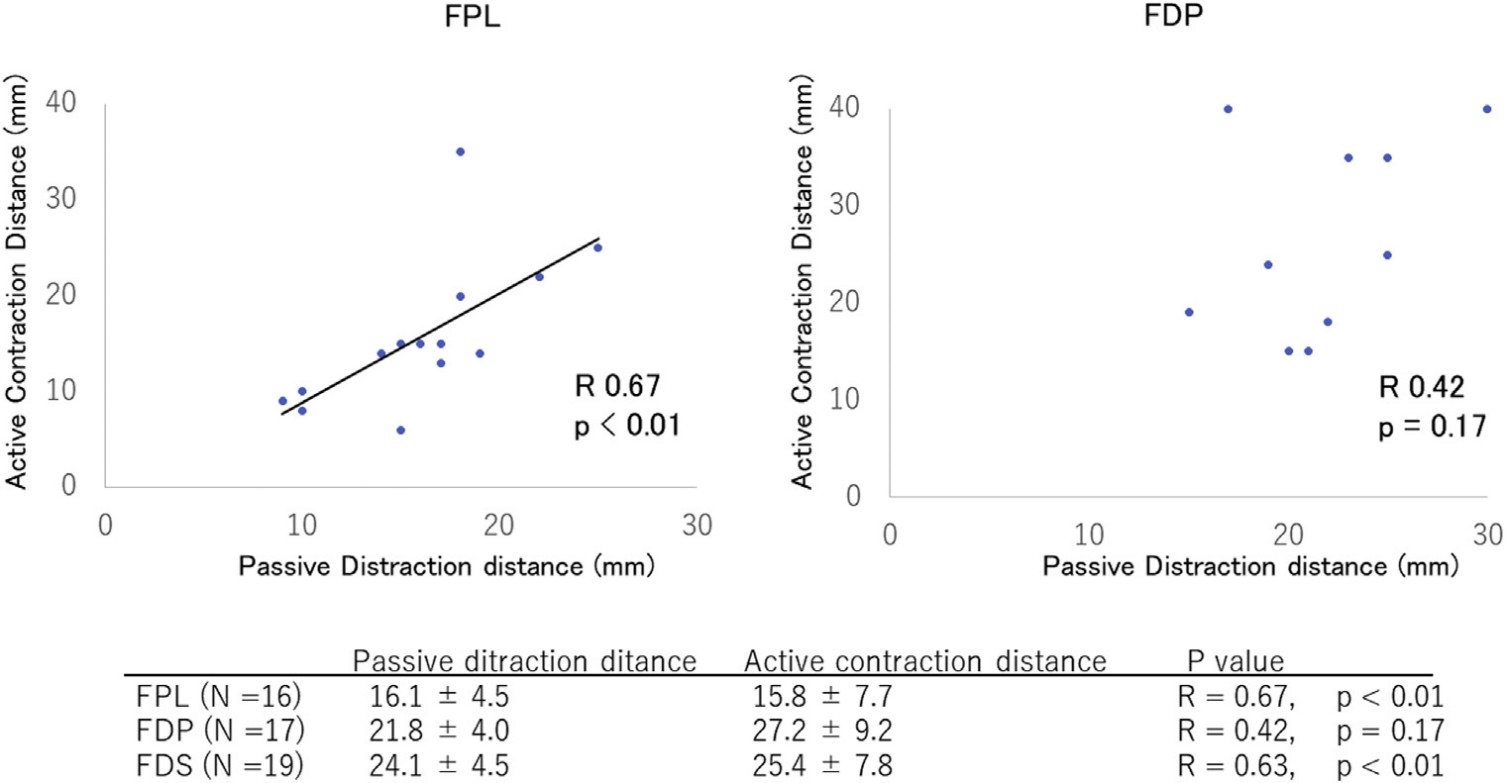
The correlation between the PDD and ACD of a ruptured musculotendinous unit.

Tendon reconstruction maintaining active finger motion during the surgery was performed in all 19 cases. The voluntary ACD of the ruptured FPL musculotendinous unit was measured in 16 cases during WALANT surgery but could not be measured in the other 3 cases because of a muscle injury or the inability to find the proximal stump. The mean PDD of the FPL musculotendinous unit was 16.1 ± 4.5 mm (range, 9–25 mm). The mean ACD of the FPL musculotendinous unit was 15.8 ± 7.7 mm (range, 4–35 mm). Passive distraction distance correlated significantly with ACD (Pearson coefficient of correlation r = 0.67; *P* < .01; Fig. 1). No significant correlation was identified between each distance and the time elapsed prior to reconstruction. The mean PDD of the donor FDS musculotendinous unit was 24.1 ± 4.5 mm (range, 15–32 mm). The mean ACD of the FDS musculotendinous unit was 25.4 ± 7.8 mm (range, 15–35 mm). Passive distraction distance correlated significantly with ACD (Pearson coefficient of correlation r = 0.63; *P* < .01).

**Figure 2:**
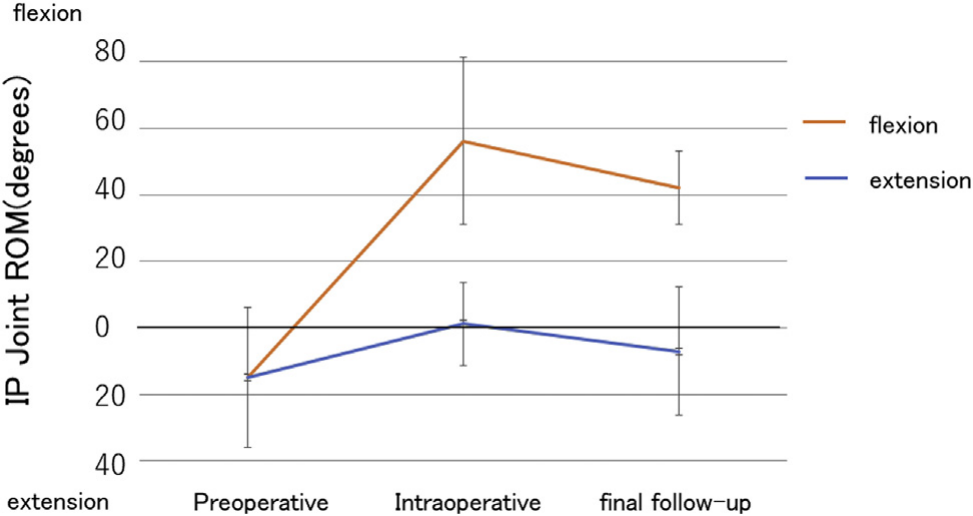
Preoperative, intraoperative, and final follow-up ranges of motion in the IP joint of the thumb in reconstruction for chronic rupture of the FPL tendon. Active flexion of the IP joint at final follow-up was significantly increased compared with that before surgery but significantly decreased compared with that during surgery.

**Figure 3:**
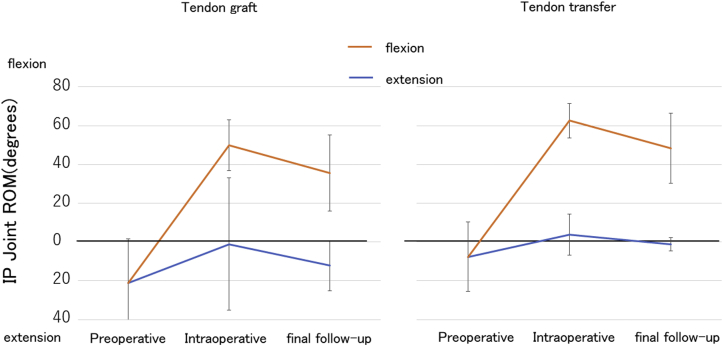
Preoperative, intraoperative, and final follow-up ranges of motion in the IP joint of the thumb between tendon grafting and tendon transfer. ROM, range of motion.

**Figure 4:**
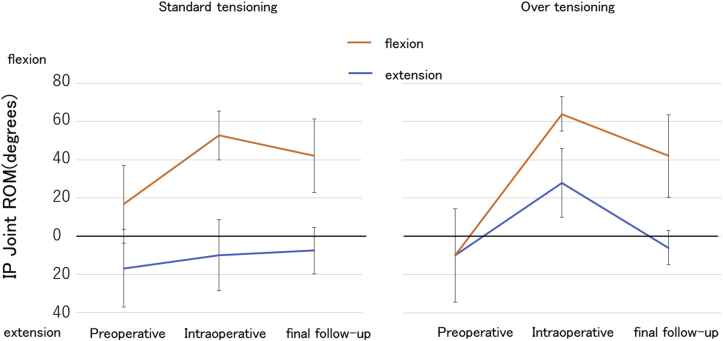
Preoperative, intraoperative, and final follow-up ranges of motion in the IP joint of the thumb between the standard-tensioning and overtensioning groups. ROM, range of motion.

Tendon grafting was performed in 10 patients with a total PDD and ACD value exceeding 30 mm, and tendon transfer was performed in 9 patients with a total PDD and ACD value less than 30 mm or with unmeasurable total PDD and ACD because of a muscle injury. In 17 cases without a joint contracture, the mean active extension and flexion of the IP joint was 14.8° and −14.8°, respectively, before the surgery; −1.2° and 56.0°, respectively, during the surgery; and 7.1° and 42.2°, respectively, at final follow-up (Table 1). The active flexion of the IP joint at final follow-up was significantly increased compared with that before the surgery but was significantly decreased compared with that during the surgery. Figure 2 shows the changes in the IP joint of the thumb. The mean TAM was 51.3° before the surgery, 114.1° during the surgery, and 103.0° at final follow-up. The mean pinch strength improved significantly from 1.4 kg before the surgery to 2.8 kg at final follow-up. The mean *Quick*DASH score improved significantly from 27.5 before the surgery to 15.8 at final follow-up. No cases showed any adverse events or required reoperation. At final follow-up, no significant differences in the IP joint range of motion, TAM, *Quick*DASH score, or pinch power were identified between tendon grafting and tendon transfer (Fig. 3) or between the standard-tensioning and overtensioning groups (Fig. 4).

**Table 1:** Functional Results of FPL Tendon Reconstruction Using the WALANT Approach

FPL Tendon Reconstruction	IP Joint ROM Ext/Flex (°) Arc (°)			TAM (% TAM)			Pinch, kg		*Quick*DASH Score	
	Preop	Intraop	Final	Preop	Intraop	Final	Preop	Final	Preop	Final
All (n = 17)	14.8/−14.8 0	−1.2/56.3 55.1	7.1/42.1 49.2	51.3 (40.8%)	114.1 (90.3%)	103 (80.8%)	1.4	2.8	27.5	15.8
Tendon graft (n = 9)	21.1/−21.1 0	1.1/50.0 51.1	12.2/36.6 48.8	50.2 (39.6%)	108.9 (87.0%)	98.3 (78.0%)	1.3	2.9	21.5	12.4
Tendon transfer (n = 8)	7.6/−7.6 0	−3.7/62.5 59.8	1.3/48.3 49.5	52.5 (42.1%)	120.0 (94.0%)	108.8 (83.9%)	1.6	2.8	33.4	19.1
Standard tensioning (n = 12)	16.8/−16.8 0	10/52.7 62.5	7.5/42.1 49.6	47.2 (38.9%)	120.8 (97.8%)	99.9 (80.3%)	1.4	2.9	23.1	13.7
Overtensioning (n = 5)	10/−10 0	−28/64 37.8	6/42 48	61.2 (45.2%)	98 (72.4%)	111.2 (81.8%)	1.4	2.8	38.5	21.0

Ext, extension; Flex, flexion; Intraop, intraoperative; Preop, preoperative; ROM, range of motion.

### FDP tendon reconstruction using the WALANT approach

Tendon reconstruction maintaining active finger motion during the surgery was performed in all the cases. The voluntary ACD of the ruptured FDP musculotendinous unit was measured in 12 cases during WALANT surgery. The mean PDD of the FDP musculotendinous unit was 21.8 ± 4.0 mm (range, 15–30 mm). The mean ACD of the FDP musculotendinous unit was 27.2 ± 9.2 mm (range, 15–40 mm). No significant correlation was identified between the distances (Pearson coefficient of correlation r = 0.42; *P* = .17; Fig. 1) or between each distance and the time elapsed prior to reconstruction. Passive distraction distance correlated significantly with ACD (Pearson coefficient of correlation r = 0.67; *P* < .01; Fig. 1).

**Figure 5:**
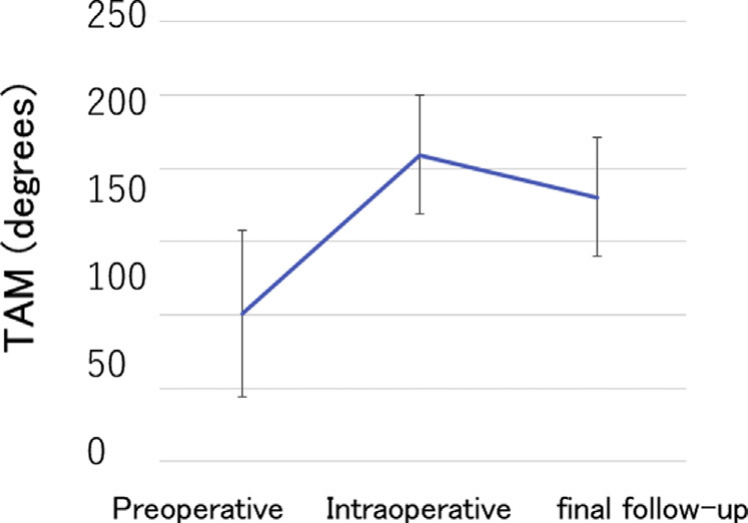
Preoperative, intraoperative, and final follow-up TAM of the affected finger in reconstruction for chronic rupture of the FDP tendon. The TAM value at final follow-up was significantly increased compared with preoperative TAM but significantly decreased compared with intraoperative TAM.

Tendon grafting was performed in 8 patients and tendon repair in 2 patients with a total PDD and ACD value greater than 40 mm, and tendon transfer was performed in 8 patients with a total PDD and ACD value less than 40 mm or with unmeasurable total PDD and ACD. In all 18 patients, the mean TAM was 101.1° before the surgery, 209.5° during the surgery, and 180.3° at final follow-up (Fig. 5). The TAM at final follow-up was significantly increased compared with that before the surgery but significantly decreased compared with that during the surgery (Fig. 5). The mean grip power improved from 9.2 kg before the surgery to 13.2 kg at final follow-up, and the mean *Quick*DASH score improved significantly from 34.1 before the surgery to 20.6 at final follow-up. At final follow-up, no significant differences in TAM, grip power, or *Quick*DASH score were evident between tendon grafting and tendon transfer or between the standard and overtensioning groups (Table 2).

**Table 2:** Functional Results of FDP Tendon Reconstruction Using the WALANT Approach

FDP Tendon Reconstruction	TAM (% TAM)			Grip Power, kg		*Quick*DASH Score	
	Preop	Intraop	Final	Preop	Final	Preop	Final
All (n = 18)	101.1 (42.1%)	209.5 (88.2%)	180.3 (75.9%)	9.2	13.2	34.1	20.6
Tendon graft (n = 8)	83.0 (35.1)	205.4 (88.6%)	178.4 (76.6%)	4.3	9.7	33.5	19.4
Tendon transfer (n = 8)	133.5 (55.6%)	228.8 (95.4%)	202.6 (84.8%)	11.2	14.2	36.0	18.6
Standard tensioning (n = 8)	47.2 (38.9%)	120.8 (97.8%)	99.9 (80.3%)	1.4	2.9	23.1	13.7
Overtensioning (n = 10)	61.2 (45.2%)	98 (72.4%)	111.2 (81.8%)	1.4	2.8	38.5	21.0

Intraop, intraoperative; Preop, preoperative.

## Discussion

In the present study, FPL and FDP tendon reconstruction could be performed in all the cases during WALANT surgery while maintaining active motion of the fingers. In tendon transfer, functional loss of the donor site could be checked during surgery, and some patients were immediately able to move their fingers on request. This allowed us to check for functional shifts in the brain. The functional outcomes of both tendon grafting and tendon transfer were good, as determined based on the evaluations of the ACD and PDD of the ruptured musculotendinous unit during WALANT surgery. This evaluation may contribute to the lack of a significant difference between tendon grafting and tendon transfer because we could select an appropriate power source that showed the muscle contraction distance. In cases in which an appropriate power source is not selected—for example, a case with good PDD but small ACD—the results of tendon grafting are likely to be poor. We have previously reported that ACD and PDD correlate positively, with almost the same values; however, we increased the number of cases and examined each tendon separately, revealing that ACD and PDD in FPL and FDS correlated significantly, whereas the correlation in FDP was weak.^20^ This may have been due to the muscle morphology of FDP. In addition, no correlation was identified between each distance and the time elapsed prior to reconstruction for any of the muscle tendons. The time elapsed prior to reconstruction did not affect ACD or PDD, which varied, probably because some patients were able to contract their muscles even after tendon rupture. In other words, the choice of tendon grafting or tendon transfer should be based on the PDD or ACD of the ruptured muscle tendon, not on the time elapsed prior to reconstruction. However, the distance at which PDD and ACD are sufficient remains unclear, and further study is needed.

The greatest advantage of tendon reconstruction during WALANT surgery was that the tension of the suture could be adjusted and that TAM could be measured during the surgery. In the past, intraoperative TAM has remained something of a “black box.” The WALANT approach reveals both intraoperative TAM and its changes. No previous reports have described intraoperative TAM in FPL and FDP tendon reconstruction. In both the procedures, the final follow-up TAM decreased from intraoperative TAM. We now know that the flexion lag was 0° during the surgery but increased over time. There was a decrease in TAM because of an extension deficit, but the effect of flexion lag was greater. This showed that the cause of flexion lag was not muscle selection or the tension of suturing, but instead, pretendinous adhesion and creep. Although some reports have described the overtensioning technique as a better option for tendon transfer, a comparison of intraoperative tension on sutured tendons between the standard-tensioning and overtensioning groups found no significant difference in the final results for flexor tendon reconstruction in this study.^24^ As a result, the best tensioning approach remains unclear, and further study is needed. A detailed review of the results of FPL tendon reconstructions showed that the tendon grafting group achieved significantly greater final extension and less final flexion than the tendon transfer group. Tendon grafting may be subject to loosening due to 2 suture sites and may be more prone to adhesions than tendon transfer. This may be because tendon grafting causes creep at the 2 suture points and forms more adhesions than tendon transfer.

The present study had some limitations that should be acknowledged. First, the investigation was based on functional outcomes in a case series, and the small number of participants decreased the strength of the statistical analyses. Additionally, the index for selecting between tendon grafting and tendon transfer was based on a past report of normal tendon excursion distance.^25^ In future studies, we would like to determine the optimal cutoff values of ACD and PDD for selecting between tendon grafting and tendon transfer, but the variability among cases will require the evaluation of many cases. Second, this method was assessed by 2 surgeons, but it was manually assessed; a device that can apply and measure quantitative force may be needed.

In conclusion, using the WALANT approach, surgeons can obtain new knowledge, select an adequate musculotendinous unit, and adjust the suture tension for the reconstruction of the muscle tendon. The WALANT approach is useful for tendon reconstruction, revealing the ACD of the muscle tendon, intraoperative TAM, functional loss at the donor site, and functional shift of the brain.

## Supplementary Data

Video 1 Measurement method for PDD and ACD. Tenolysis of the ruptured tendon was performed until the muscle-tendon junction was observed. The proximal stump of the ruptured tendon was mobilized as much as possible, and the PDD from the resting position to the maximum distal point was measured. Then, the patients were asked to flex and extend their thumbs with maximum power, and the voluntary ruptured musculotendinous unit contraction to the proximal side was observed. The ACD from the resting position to the maximum proximal point was measured.

Video 2 Total active motion before reconstruction for chronic rupture of the FPL tendon.

Video 3 Intraoperative TAM during WALANT surgery.

Video 4 Total active motion at final follow-up.
